# Novel Biomarkers Differentiating Viral from Bacterial Infection in Febrile Children: Future Perspectives for Management in Clinical Praxis

**DOI:** 10.3390/children8111070

**Published:** 2021-11-20

**Authors:** Samuel Rhedin, Kristina Elfving, Anna Berggren

**Affiliations:** 1 Department of Medical Epidemiology and Biostatistics, Karolinska Institutet, s-171 65 Stockholm, Sweden; Samuel.rhedin@ki.se; 2 Sachs’ Children and Youth Hospital, Södersjukhuset, s-118 61 Stockholm, Sweden; 3 Institution of Medicine, School of Public Health, Sahlgrenska Academy, University of Gothenburg, s-411 24 Gothenburg, Sweden; kristina.elfving@gu.se; 4 Department of Pediatrics, The Queen Silvia Children’s Hospital, s-413 45 Gothenburg, Sweden; 5 Infectious Disease Unit, Department of Medicine Solna, Karolinska Institutet, Karolinska University Hospital, s-171 65 Stockholm, Sweden; 6 Research and Development, Norrtälje Hospital, s-761 29 Norrtälje, Sweden

**Keywords:** biomarkers, pediatric infectious diseases

## Abstract

Differentiating viral from bacterial infections in febrile children is challenging and often leads to an unnecessary use of antibiotics. There is a great need for more accurate diagnostic tools. New molecular methods have improved the particular diagnostics of viral respiratory tract infections, but defining etiology can still be challenging, as certain viruses are frequently detected in asymptomatic children. For the detection of bacterial infections, time consuming cultures with limited sensitivity are still the gold standard. As a response to infection, the immune system elicits a cascade of events, which aims to eliminate the invading pathogen. Recent studies have focused on these host–pathogen interactions to identify pathogen-specific biomarkers (gene expression profiles), or “pathogen signatures”, as potential future diagnostic tools. Other studies have assessed combinations of traditional bacterial and viral biomarkers (C-reactive protein, interleukins, myxovirus resistance protein A, procalcitonin, tumor necrosis factor-related apoptosis-inducing ligand) to establish etiology. In this review we discuss the performance of such novel diagnostics and their potential role in clinical praxis. In conclusion, there are several promising novel biomarkers in the pipeline, but well-designed randomized controlled trials are needed to evaluate the safety of using these novel biomarkers to guide clinical decisions.

## 1. Introduction

Acute infections represent a major cause of morbidity and mortality in children around the world, predominately in small children living in low- and middle-income countries [1]. However, also in high-income countries, infection is one of the most common reasons for seeking medical care at pediatric emergency units and thus posing a significant burden on health care systems and causing large economic consequences both for the family and society. When a child enters the emergency unit with an infection, it is of great importance to identify the etiology for further clinical management. The vast majority of pediatric infections are viral, but in young infants below three months of age, the likelihood of bacterial etiology is increased, and bacterial infections that can be potentially lethal can be detected in up to 8–14% in this group [2,3]. In many cases, fever is the only symptom present, making it a clinical challenge to differentiate self-resolving viral infections from serious bacterial infections.

Because of the difficulties in differentiating viral from bacterial infections, ill-appearing children suffer from extensive invasive testing and unnecessary usage of antibiotics. This is a great concern, as inappropriate usage of antibiotics contributes to the emerging threat of antimicrobial resistance but also disturbs the microbial flora of the gut, leading to potentially large negative consequences for the infant [4,5]. Therefore, there is a great need for more specific and reliable diagnostic tools for the identification of antibiotic-requiring bacterial infections in children.

Bacterial cultures from sterile sites are still considered the golden standard for the establishment of bacterial etiology, but the sensitivity is low, and the results may take several days [6]. For respiratory viral detection, molecular-based methods such as PCR, with increased sensitivity as compared with historical virological methods, have widely been introduced during the last decade. However, the interpretation of the results is complicated by the fact that certain respiratory viruses have been detected in up to 40% of asymptomatic children [7]. It is also a challenge to obtain representative specimens from the source of infection in children, such as from the lower respiratory tract [6]. Given the limitations with current microbiological testing in children, there has lately been an increased interest in the host’s response to invading pathogens, with the aim of identifying novel biomarkers that accurately differentiate between viral and bacterial etiology. In this review, we discuss such novel biomarkers and their potential implication in clinical praxis.

## 2. Methods

The aim of this narrative review was to present an overview of studies on novel biomarkers for the differentiation between viral and bacterial etiology in the febrile child presenting at the emergency department (Table 1). The focus was on studies published within the last 10 years, and articles were identified by searches in PubMed using medical subject headings (MeSH) as follows (Biomarker AND (fever (MeSH) OR sepsis (MeSH) OR neonatal sepsis (MeSH)) AND (child (MeSH) OR child, preschool [MeSH] OR infant (MeSH]) AND (“2010” (Date—Publication): “2021” (Date—Publication)) as well as through cross-references.

## 3. Single Biomarkers and Combined Tests That Distinguish Viral and Bacterial Etiology

### 3.1. Routine Biomarkers

White blood counts (WBC) have been used for decades to identify infants with severe bacterial infection, normally by using a threshold level of 15,000 cells/mm^3^ [8,9]. However, in the post-vaccine era, the bacterial spectrum has changed [10] and the usefulness of WBC as a predictor for bacterial infections in infants has been questioned, as most studies have shown a low predictive value [10,11,12,13,14,15,16]. Only a few studies have in the post-vaccine era investigated the performance of WBC as a predictor of bacterial infections in children older than 12 months, with the same conclusions as with younger infants [10,17,18,19]. C-reactive protein (CRP) is a commonly used biomarker for infection worldwide. Blood levels increase at time of infection, but elevated levels are also seen in other diseases, such as inflammatory disorders, cancer and trauma [20]. In a systematic review, Sanders et al. could show that CRP gave moderate information in both ruling in and out serious bacterial infections in children with fever in an outpatient setting [21]. In later studies, the same results have been confirmed for CRP, both as a single marker [22,23,24] but also together with other markers in clinical algorithms, such as the “step-by-step” approach [25]. The diagnostic accuracy for discriminating viral from bacterial etiologies is however limited, especially in early stages [21,26,27]. Procalcitonin (PCT) has, in most studies, been shown to be a superior biomarker as compared with CRP for the differentiation between infectious and non-infectious inflammation, and blood levels increase more rapidly [28,29,30,31,32]. However, the specificity for distinguishing between viral and bacterial infections is limited, particularly in children <21 days [31,33,34]. Currently, the use of PCT is mostly evaluated for ruling out severe bacterial infections in infants in combination with other microbiological findings and clinical signs [31,34,35,36,37,38], but PCT also has potential utility in the management of febrile urinary tract infections, pneumonia and non-infectious inflammatory syndromes [28,39,40]. PCT increases physiologically in newborns during the first days of life [38].

### 3.2. Interleukins as Biomarkers for Sepsis and Bacterial Infection

Interleukins (ILs) mediate communication between cells and are pivotal in the pro- and anti-inflammatory early immune responses to infections. The focus of ILs’ role as an infection biomarker has been mostly as a potential biomarker for sepsis and bacterial infections, and studies during the last decade on children in a post-neonatal setting are few [15,41,42,43,44,45,46]. IL6 is the most studied IL and has been shown to have a potential prognostic value in children with sepsis. The usefulness of IL6 can be increased in combination with other biomarkers [43,47,48]. A challenge with ILs is the variations in serum concentrations at different time points. In addition, the lack of a commonly used definition of pediatric sepsis makes translation of findings between studies difficult, highlighting the need for future studies with well-defined study cohorts.

### 3.3. Viral Biomarkers and Combination Tests

While most commercially available biomarkers have been focused on the identification of serious bacterial infection (SBIs), there is currently an increasing interest in viral biomarkers. As novel antiviral therapeutic possibilities arise and new vaccines targeting viruses are developed, the accurate identification of children with viral infections will be pivotal. Moreover, given the complexity of the host immune response to infections and the increasingly recognized importance of viral–bacterial interactions, it is likely unrealistic to think that one single biomarker would be able to accurately identify children with antibiotic-requiring bacterial infections [49,50]. Hence viral biomarkers can add further value in the differentiation between viral and bacterial etiology if analyzed in combination with other bacterial or inflammatory biomarkers.

#### 3.3.1. Myxovirus Resistance Protein A

Myxovirus resistance protein A (MxA) is a small peptide with antiviral activity that is produced in a variety of immune cells and is rapidly upregulated by interferon signaling [51]. MxA levels have been shown to be higher in children with viral etiology, as compared with bacterial etiology in children with febrile illness, and therefore MxA levels are a promising biomarker for differentiating between these two etiologies [52]. In addition, MxA levels assist in the distinction between active infection and asymptomatic detection in children with respiratory symptoms, which is a common clinical problem when interpreting PCR data of certain respiratory viruses in children [7,51]. A commercial point-of-care combination test of MxA and CRP is approved for use in a number of European countries but has so far mostly been evaluated in an adult population, and thus more studies on MxA in children are needed [53,54].

#### 3.3.2. Tumor Necrosis Factor-Related Apoptosis-Inducing Ligand

Tumor necrosis factor-related apoptosis-inducing ligand (TRAIL) is another viral biomarker that historically has been used as a biomarker for cardiovascular autoimmune disease but that has lately been identified as a promising viral biomarker [55,56]. TRAIL is included in a United States Food and Drug Administration (FDA)-approved commercial combination test together with CRP and interferon gamma-induced protein 10 [57,58]. This combination test has been shown to be superior to PCT in terms of accuracy for distinguishing bacterial and viral infections in two external validation studies of children with febrile illness [57,58,59]. However, in the largest study, the performance of CRP was almost as good (area under the curve 0.89 vs 0.90) as the commercial test, underscoring the methodological challenges in this kind of study, as the imperfect biomarker CRP is often used directly or indirectly as reference standard [58,60].

### 3.4. Gene Expression Profiling 

Since the development of microarrays, the possibility of studying the pathogen–host interaction has entered a new era [61,62]. Ribonucleic acid (RNA) can be isolated from the peripheral whole blood that constitutes a majority of white blood cells. Consequently, the host’s immune response, as reflected by the gene expression signals from the white blood cells, can be studied in detail. These techniques have the potential to be used both as pathogen-specific diagnostic tools but also as tools to discriminate an active infection from asymptomatic detection.

#### 3.4.1. Pathogen-Specific Signatures in Children

In an attempt to identify discriminative transcriptional signatures in children with an acute infection, gene expression profiles from acutely infected children with defined infections have been evaluated [63,64]. Hereby, researchers have been able to identify sets of differently expressed genes that not only correctly distinguish febrile virus-positive children not only from afebrile controls but also from asymptomatic afebrile children with the same virus present [64]. With the same approach, bacterial infections have been distinguished from viral infections [63,64]. In addition, a number of studies have been able to define pathogen/disease-specific signatures with different etiologies in children [65,66,67,68,69,70]. However, to be useful as a diagnostic tool in the clinic, microarrays and RNA sequencing-based techniques need to be converted to rapid point-of-care platforms. To succeed with that conversion, the number of classifier genes must be reduced to a minimum. Recently, researchers identified a 2-script-Host-RNA signature that differentiated between viral and bacterial infections in children with a sensitivity of 100% and a specificity of 96.4%; in addition, it was successfully tested in children with inflammatory disease [71]. Since then, the signature was validated as being able to correctly distinguish viral from bacterial etiology in children with gastroenteritis [72], and a qPCR expression assay detecting these two genes has successfully been set up, in addition to a recent point-of-care platform yields results within 25 min [73,74].

Another approach to finding a suitable diagnostic tool to be used in the clinic is to use multi-cohort data accessible via public platforms [75,76,77,78,79,80]. By using a multi-data cohort approach including both adult and pediatric patients, and also animals in one of the studies, two research groups were able to identify a 7- and 4- script-Host RNA signature that discriminated viral from bacterial infection and viral from non-infective inflammation with high accuracy [75,76]. These gene expression-based diagnostic approaches are promising and have been proven superior to other biomarkers, such as white blood counts and PCT [63,64,78], but further evaluation in different patient cohorts is needed, especially since a recent publication could show that gene expression profiling was not feasible in children under treatment for cancer and presenting with febrile neutropenia [81].

#### 3.4.2. Future Ways to Diagnose Coinfections and Distinguish Asymptomatic from Symptomatic Infection

Another aspect to consider in the clinic is the translation of positive viral PCR findings, as certain respiratory viruses frequently are detected in asymptomatic children [7]. In addition, studies have shown that viral and bacterial coinfections are common in children with different infections, such as pneumonia and otitis media. [82,83]. These two aspects are of special importance to consider when handling patients with increased risk of bacterial infections, such as immunosuppressed children, where the judgment of when to initiate and stop antibiotics could be difficult. Human rhinovirus is the most common virus causing common cold symptoms, but it is also frequently detected in asymptomatic children [7,84]. When using gene expression analysis, researchers were able to distinguish asymptomatic rhinovirus infection from symptomatic illness [84]. In addition, symptomatic human herpesvirus-6 infections in children were possible to distinguish from asymptomatic infection but impossible to distinguish from controls without an infection present [64]. These findings indicate that transcriptional signals can be useful in the discrimination of symptomatic and asymptomatic infections. Indeed, recent studies of nasal swabs from patients with respiratory infection have identified sets of genes that are concordant with an active viral respiratory infection [85,86,87]. Co-infections are difficult to define and have not been addressed in the majority of the published gene expression-based studies. However, two studies have evaluated transcriptional signals in smaller cohorts of patients with co-infections with promising results, but larger cohorts are needed [77,78].

## 4. Future Perspectives

To reduce the morbidity and mortality and improve the usage of antibiotics, there is an urgent need for better diagnostic tools in the clinic for children presenting with acute infections. An ideal biomarker should not only identify serious infections but also accurately exclude non-infectious causes of inflammation to be able to guide the clinician in the important decision of whether or not to prescribe antibiotics.

Viral biomarkers and combination tests have the potential to improve the accuracy of identifying bacterial infections as compared with old inflammatory single biomarkers, but the lack of a good reference standard for bacterial infections makes it difficult to properly evaluate the performance. Furthermore, viral–bacterial co-infections remain a challenge, and evidence from microbiota studies suggests that mixed viral–bacterial infections are likely underestimated [49,88]. However, from a clinical point of view, it is more important to identify antibiotic-requiring bacterial infections rather than to determine the exact etiology of each infectious episode, as many bacterial or mixed viral–bacterial infections are in fact self-limiting.

So far, there has not been a consensus regarding the reference standard for bacterial infections, and both expert panels and algorithm-based approaches have been used [57,89]. This makes it difficult to compare the findings from different studies. Recently, the algorithm used for classification of microbiological etiology in the Personalized Risk Assessment in Febrile Children to Optimize Real-life Management across the European Union (PERFORM) consortium was validated in five independent cohorts of previous biomarker studies. By using the PERFORM classification, the accuracy of the studied biomarkers increased as compared with the previously used classification of SBI versus non-SBI. This framework could potentially serve as a novel standard for the classification of etiology for future biomarker studies [90].

Diagnostic methods based on gene expression are promising and have been shown to successfully distinguish viral from bacterial infections with high sensitivity and specificity and also to distinguish symptomatic from asymptomatic infections [71,84]. There is also evidence that co-infections can be correctly diagnosed [78]. However, as there is diversity in gene expression signaling between sexes and patients with different genetic backgrounds and also on an individual level, it is of utmost importance to validate these methods in different patient cohorts [91]. Even if the findings so far have been reproducible in children of different genetic backgrounds and for various pathogens [72,73], these findings still need to be investigated in groups of children with different underlying conditions, especially conditions mimicking those of an infection, such as asthma and inflammatory disorders and in immune-suppressed children.

With increasing evidence from observational studies, it appears as if the next natural step to push the field forward is to assess the safety of decision-making guidance regarding antibiotic treatment based on different novel biomarkers and combination tests in well-designed randomized controlled trials of children with specific diagnoses and with different genetic backgrounds. However, it is also important to recognize that the implementation of a novel diagnostic test might result in increased antibiotic treatment if it identifies self-limiting bacterial or mixed viral–bacterial infections that were previously undiagnosed [34].

Another major challenge of novel diagnostic tests is the turnaround time. For ideal usage at the emergency department, the results of a point-of-care test should preferably be available within an hour. Diagnostic platforms are under construction, and future focus should be on further development of cheap point-of-care platforms with a short turnaround time [73,74,78,92].

## 5. Conclusions

There is a great need for improved diagnostic tests that accurately distinguish between viral and bacterial etiology of febrile children. Several promising novel biomarkers are in the pipeline, but the lack of a reference standard for microbiological etiology is hampering the evaluation of these novel tests, while another great challenge is the need for a short turnaround time. To further push the field forward, well-designed randomized controlled trials are needed to evaluate the safety of decision-making guidance for antibiotic treatment based on these novel biomarkers.

## Figures and Tables

**Table 1:** Overview of biomarkers included in the review.

Type	Biomarker	Comment
Routine biomarkers	CRP	Widely introduced in the clinic. Limited specificity. Delayed increase in blood.
	WBC	Low specificity.
	PCT	Rapid increase in blood. Mostly evaluated for sepsis, pneumonia and urinary tract infection.
Bacterial, inflammatory markers	Interleukins	Rapid increase in blood. Mostly evaluated for sepsis.
Viral biomarkers	MxA, TRAIL	Promising as complement to bacterial biomarkers.
Blood mRNA biomarkers	mRNA transcripts	Promising. Not yet commercially available.

Abbreviations: CRP, C-reactive protein; mRNA, messenger ribonucleic acid; MxA, Myxovirus resistance protein A; PCT, procalcitonin; TRAIL, tumor necrosis factor-related apoptosis-inducing ligand; WBC, white blood cell count.
